# DELETION OF THROMBOSPONDIN-1 PRESERVES HEMATOPOIETIC STEM CELL HEALTHSPAN DURING AGING

**DOI:** 10.1093/geroni/igac059.2664

**Published:** 2022-12-20

**Authors:** Pradeep Ramalingam, Michael Gutkin, Michael Poulos, Jason Butler

**Affiliations:** University of Florida, Gainesville, Florida, United States; Hackensack University Medical Center, Nutley, New Jersey, United States; Hackensack University Medical Center, Nutley, New Jersey, United States; Hackensack University Medical Center, Nutley, New Jersey, United States

## Abstract

Aging is associated with defects within blood stem cells, termed hematopoietic stem cells (HSC), including a loss of their self-renewal potential and a skewed differentiation towards myeloid lineages at the expense of lymphoid cells. Collectively, these HSC defects manifest as anemias, poor response to vaccines and an increased incidence of myeloid neoplasms in older adults. Unlike other somatic stem cells, aged HSCs have been shown to be refractory towards established anti-aging interventions including caloric restriction, exercise, parabiosis and plasma transfer. Thrombospondin-1 (TSP1) was initially discovered as an anti-angiogenic molecule, and recent studies have identified that TSP1 promotes age-related pathologies including chronic inflammation, reactive oxygen species (ROS) generation, and mitochondrial dysfunction. Notably, each of these TSP-1 regulated processes have been shown to critically influence HSC biology, particularly in the context of aging. However, whether TSP-1 directly regulates HSC activity remains unexplored. Here, we sought to determine whether TSP-1 is essential for HSC development, and whether blocking TSP1 signaling could ameliorate age-related HSC defects. Utilizing murine models, we demonstrate that TSP-1 is dispensable for normal HSC development and hematopoiesis. We show that deletion of TSP-1 is sufficient to preserve HSC fitness during aging, as evidenced by preservation of youthful self-renewal potential and balanced lineage reconstitution during serial HSC transplantation assays. Mechanistically, we identify that TSP-1 adversely impacts mitochondrial metabolism within HSCs, and show that loss of TSP-1 prevents the age-related decline in HSC mitochondrial membrane potential. Our findings identify TSP-1 as a pro-geronic factor that can be targeted to preserve HSC healthspan.

